# Supplementary material for: Auditory brainstem responses as a biomarker for cognition
Source: Commun Biol. 2024 Dec 19;7:1653. doi: 10.1038/s42003-024-07346-4 (PMC11659319; doi:10.1038/s42003-024-07346-4)

Supplementary Materials for "Auditory Brainstem Responses as a Biomarker for Cognition"

Yasmeen Hamza[1,2,*], Ye Yang[1], Janie Vu[1], Antoinette Abdelmalek[1], Mobina Malekifar[1], Carol A. Barnes[3], Fan-Gang Zeng[1,*]

[1]Center for Hearing Research, Otolaryngology-Head and Neck Surgery, University of California Irvine, Irvine, California, USA
[2] Institute of Sound and Vibration Research, School of Engineering, University of Southampton, Southampton, UK
[3]Psychology, Neurology and Neuroscience, and Evelyn F. McKnight Brain Institute, University of Arizona, Tuscan, Arizona, USA

The PDF file includes:

Supplementary Table 1. The Association between cognition and ABR measures.
Supplementary Table 2. The Association of PTA and ABR measures.
Supplementary Table 3. The Association between the ABR-V latency and Cognitive Domains.
Supplementary Table 4. The Association between the ABR-V Amplitude and Cognitive Domains.
Supplementary Table 5. The Association between the hearing level or PTA and Cognitive Domains.
Supplementary Figure 1. The area-under-curve (AUC) using ABR wave V parameters to predict age-unadjusted and adjusted cognitive performance.
Supplementary Figure 2. Area-under-curve (AUC) analysis using 5-fold cross validation.
Supplementary Figure 3. Area-under-curve (AUC) analysis using 10-fold cross validation.

***Please send correspondence to:**
Yasmeen Hamza
Room 4023, Building 13
University Road Southampton, SO17 1BJ
Email: y.hamza@soton.ac.uk
or
Fan-Gang Zeng
110 Medical Science E
Irvine, CA 92697
Email: fzeng@uci.edu

Supplementary Table 1. The Association between cognition and ABR measures

	Univariate			Age-Adjusted		
Independent Variable	B	CI	p	B	CI	p
Wave I Latency	-0.023	-0.137– 0.091	0.690	0.008	-0.075– 0.091	0.851
Wave I Amplitude	0.043	-0.071– 0.156	0.460	-0.020	-0.104– 0.063	0.632
Wave V Latency	-0.212	-0.322– -0.101	<0.0001*	-0.101	-0.186– -0.016	0.021*
Wave V Amplitude	0.288	0.183– 0.392	<0.0001*	0.110	0.018– 0.202	0.020*
I-V Latency Difference	-0.172	-0.281– -0.062	0.002*	-0.094	-0.176– -0.011	0.026*
V/I Amplitude Ratio	0.088	-0.024– 0.200	0.124	0.068	-0.014– 0.151	0.104

Supplementary Table 2. The Association of PTA* and ABR measures

	Univariate			Age-Adjusted		
Dependent Variable	B	CI	p	B	CI	P
Wave I Latency	-0.018	-0.201– 0.166	0.846	-0.071	-0.197– 0.056	0.271
Wave I Amplitude	-0.125	-0.308– 0.058	0.178	-0.004	-0.133– 0.126	0.953
Wave V Latency	0.122	-0.063– 0.307	0.193	-0.059	-0.190– 0.072	0.374
Wave V Amplitude	-0.345	-0.522– -0.168	<0.0001*	-0.035	0.178– 0.107	0.624
I-V Latency Difference	0.115	-0.069– 0.300	0.219	-0.003	-0.133– 0.128	0.970
V/I Amplitude Ratio	-0.067	-0.254– 0.121	0.483	-0.059	-0.190– –0.072	0.375

*PTA was log-transformed before z-scoring to meet the assumptions of linear regression.

Supplementary Table 3. The Association between the ABR-V latency and Cognitive Domains

	Univariate			Multivariate		
Dependent Variable	B	CI	p	B	CI	P
Composite cognitive	-0.212	-0.322– -0.101	<0.0001*	-0.101	-0.186– -0.016	0.021*
Word Learning	-0.299	-0.474– -0.123	0.001*	-0.200	-0.370– -0.031	0.021*
Delayed recall	-0.302	-0.478– -0.127	0.001*	-0.197	-0.364– -0.030	0.021*
Word Recognition	N/A	N/A	N/A	N/A	N/A	N/A
Animal Fluency	-0.285	-0.461– -0.108	0.002*	-0.200	-0.374– -0.026	0.025*
TMT A (Executive)	-0.128	-0.310– 0.055	0.168	0.007	-0.158– 0.172	0.935
TMT B (Executive)	-0.229	-0.408– -0.050	0.013*	-0.103	-0.268– 0.061	0.216
4 MT accuracy (spatial)	-0.035	-0.219– 0.149	0.708	0.100	-0.067– 0.267	0.237
4 MT RT (spatial)	-0.088	-0.271– 0.095	0.342	-0.011	-0.194– 0.172	0.909
Visual Discrimination	-0.185	-0.366– -0.004	0.045*	-0.070	-0.241– 0.100	0.415
SDMT (Attention/ working memory)	-0.299	-0.475– -0.124	0.001*	-0.126	-0.264– 0.011	0.071

N/A: Assumptions of homoscedasticity and normality of errors were not met due to a ceiling effect in the word recognition test.

Supplementary Table 4. The Association between the ABR-V Amplitude and Cognitive Domains

	Univariate			Multivariate		
Dependent Variable	B	CI	p	B	CI	P
Composite cognitive	0.288	0.183– 0.392	<0.0001*	0.110	0.018– 0.202	0.020*
Word Learning	0.355	0.183– 0.527	<0.001*	0.206	0.023– 0.390	0.028*
Delayed recall	0.276	0.099– 0.452	0.003*	0.091	-0.093– 0.275	0.329
Word Recognition	N/A	N/A	N/A	N/A	N/A	N/A
Animal Fluency	0.499	0.340– 0.658	<0.001*	0.415	0.239– 0.591	<0.001*
TMT A (Executive)	0.294	0.118– 0.469	0.001*	0.083	-0.095– 0.261	0.359
TMT B (Executive)	0.304	0.129– 0.479	0.001*	0.098	-0.080– 0.276	0.280
4 MT accuracy (spatial)	0.151	-0.031– 0.333	0.103	0.083	-0.264– 0.097	0.363
4 MT RT (spatial)	0.144	-0.038– 0.326	0.119	0.014	-0.183– 0.212	0.887
Visual Discrimination	0.306	0.131– 0.481	0.001*	0.129	-0.054– 0.312	0.165
SDMT (Attention/ working memory)	0.462	0.299– 0.625	<0.001*	0.191	0.045– 0.337	0.011*

N/A: Assumptions of homoscedasticity and normality of errors were not met due to a ceiling effect in the word recognition test.

Supplementary Table 5. The Association between the hearing level or PTA and Cognitive Domains

	Univariate			Multivariate		
Dependent Variable	B	CI	p	B	CI	P
Composite cognitive	-0.021	-0.027– -0.014	<0.001*	-0.003	-0.010– 0.004	0.385
Word Learning	-0.022	-0.033– -0.011	<0.001*	-0.008	-0.022– 0.007	0.298
Delayed recall	-0.014	-0.026– -0.003	0.017*	0.009	-0.005– 0.023	0.196
Word Recognition	N/A	N/A	N/A	N/A	N/A	N/A
Animal Fluency	-0.019	-0.030– -0.008	0.001*	-0.005	-0.020– 0.010	0.484
TMT A (Executive)	-0.022	-0.033– -0.011	<0.001*	-0.001	-0.015– 0.012	0.835
TMT B (Executive)	-0.029	-0.039– -0.018	<0.001*	-0.013	-0.027– -0.000	0.054
4 MT accuracy (spatial)	-0.022	-0.033– -0.011	<0.001*	-0.003	-0.017– 0.011	0.658
4 MT RT (spatial)	-0.013	-0.024– -0.001	0.033*	0.000	-0.015– 0.015	0.995
Visual Discrimination	-0.022	-0.033– -0.011	<0.001*	-0.005	-0.019– 0.009	0.461
SDMT (Attention/ working memory)	-0.034	-0.044– -0.024	<0.001*	-0.009	-0.020– 0.003	0.138

N/A: Assumptions of homoscedasticity and normality of errors were not met due to a ceiling effect in the word recognition test.

Supplementary Figure 1. The area-under-curve (AUC) using ABR wave V parameters to predict age-unadjusted (A, B) and adjusted (C,D) cognitive performance. A. The receiver-operating-characteristic (ROC) curve using >6.42-ms latency as the criterion to detect low cognitive performers. The open orange circle represents the optimal sensitivity and specificity outcomes. **B**. The ROC using >0.39-μV latency to detect high cognitive performers. The open purple circle represents the optimal sensitivity and specificity outcomes. **C**. The ROC using >6.24-ms latency as the criterion detected low cognitive performers below 3-percentile with 71% sensitivity and 79% specificity (solid orange circle). **D.** The ROC using >0.25-μV amplitude as the criterion detected high cognitive performers above 93-percentile with 84% sensitivity and 71% specificity (solid purple circle).

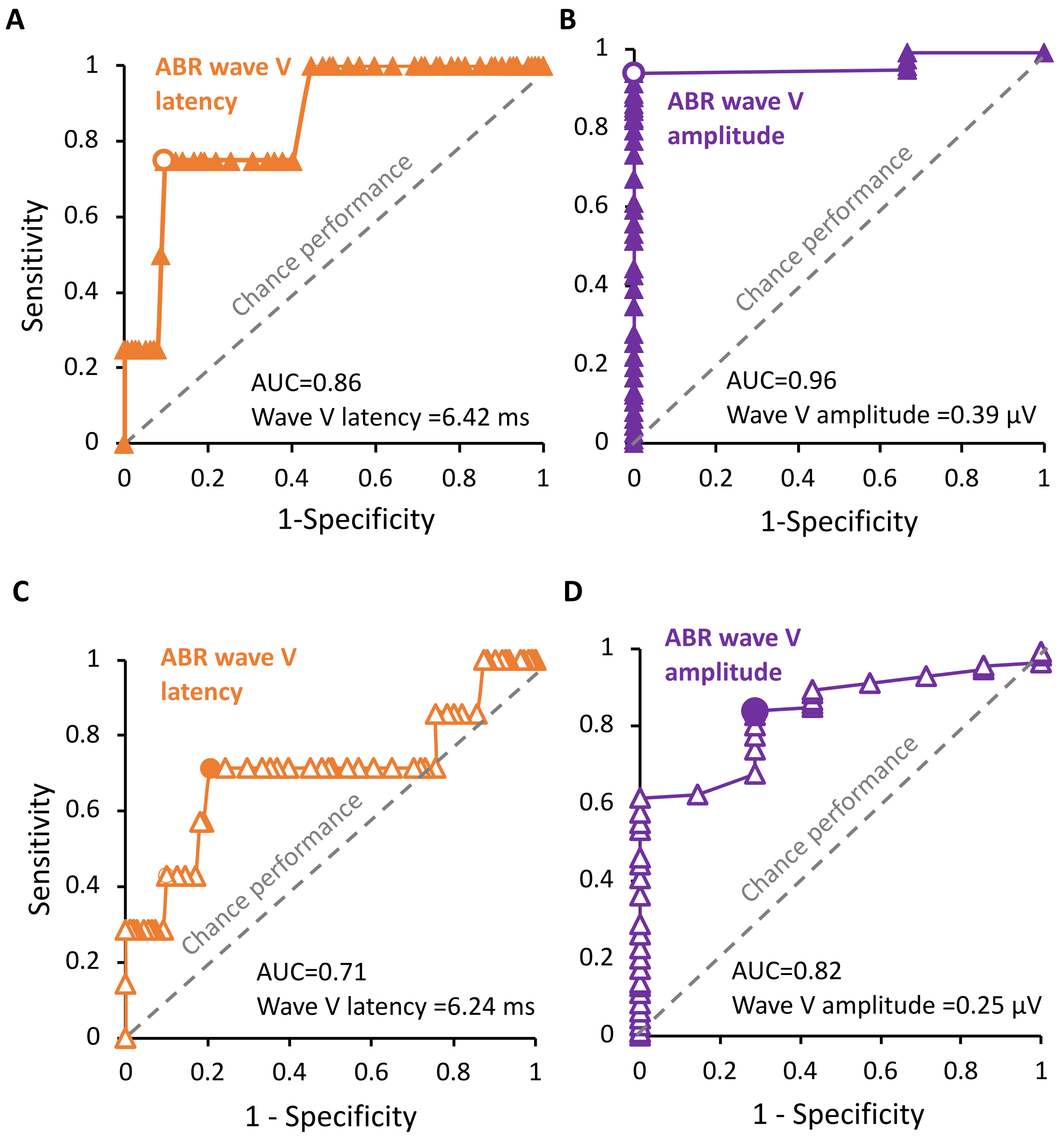

Supplementary Figure 2. Area-under-curve (AUC) analysis using 5-fold cross validation. Same as Fig. 5 except that the AUC vs cognitive performance function was generated using 5-fold cross validation. **A.** Age unadjusted cognitive performance (x-axis). **B.** Age adjusted cognitive performance (x-axis).

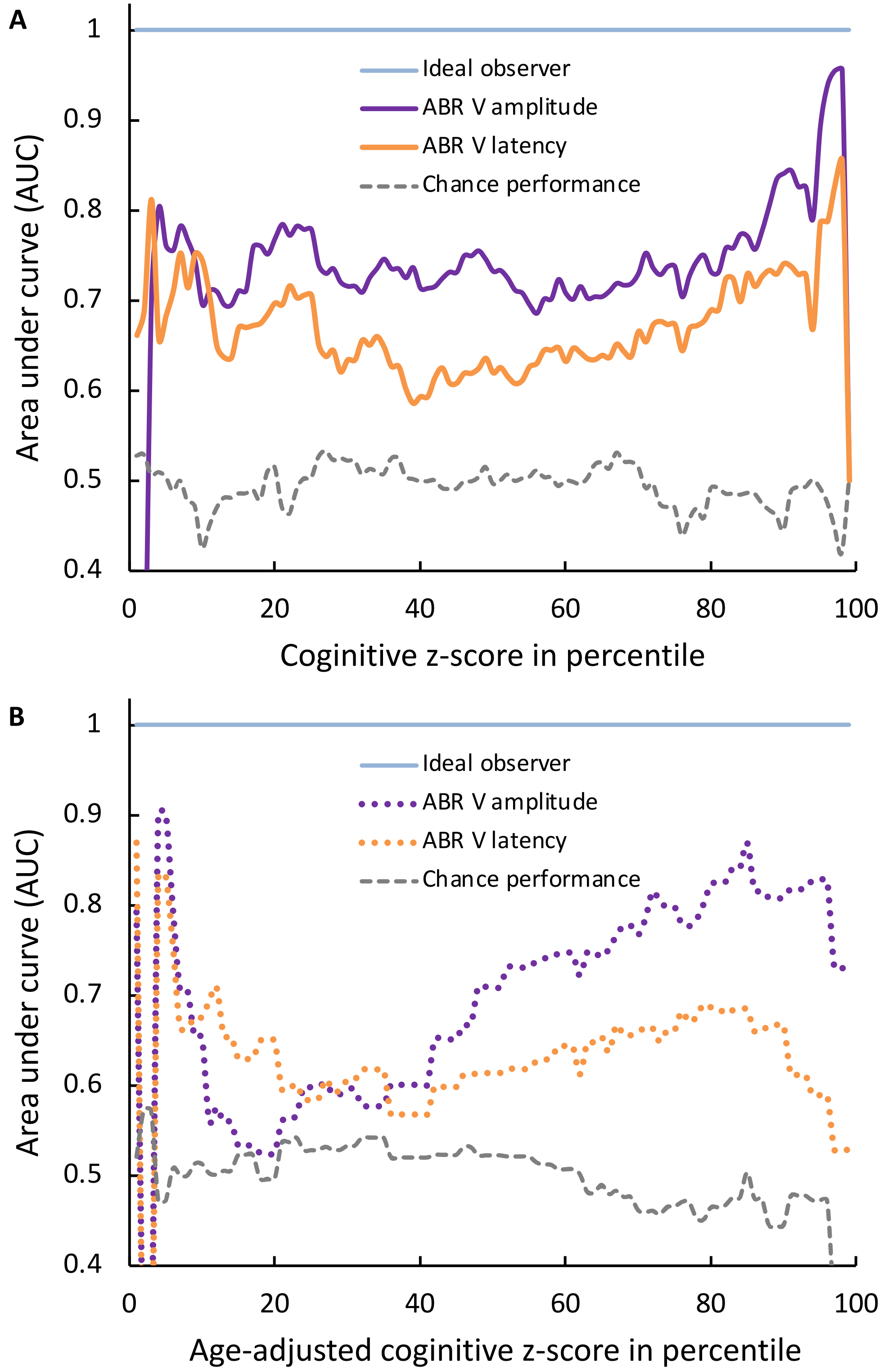

Supplementary Figure 3. Area-under-curve (AUC) analysis using 10-fold cross validation. Same as Fig. 5 except that the AUC vs cognitive performance function was generated using 10-fold cross validation. **A.** Age unadjusted cognitive performance (x-axis). **B.** Age adjusted cognitive performance (x-axis).

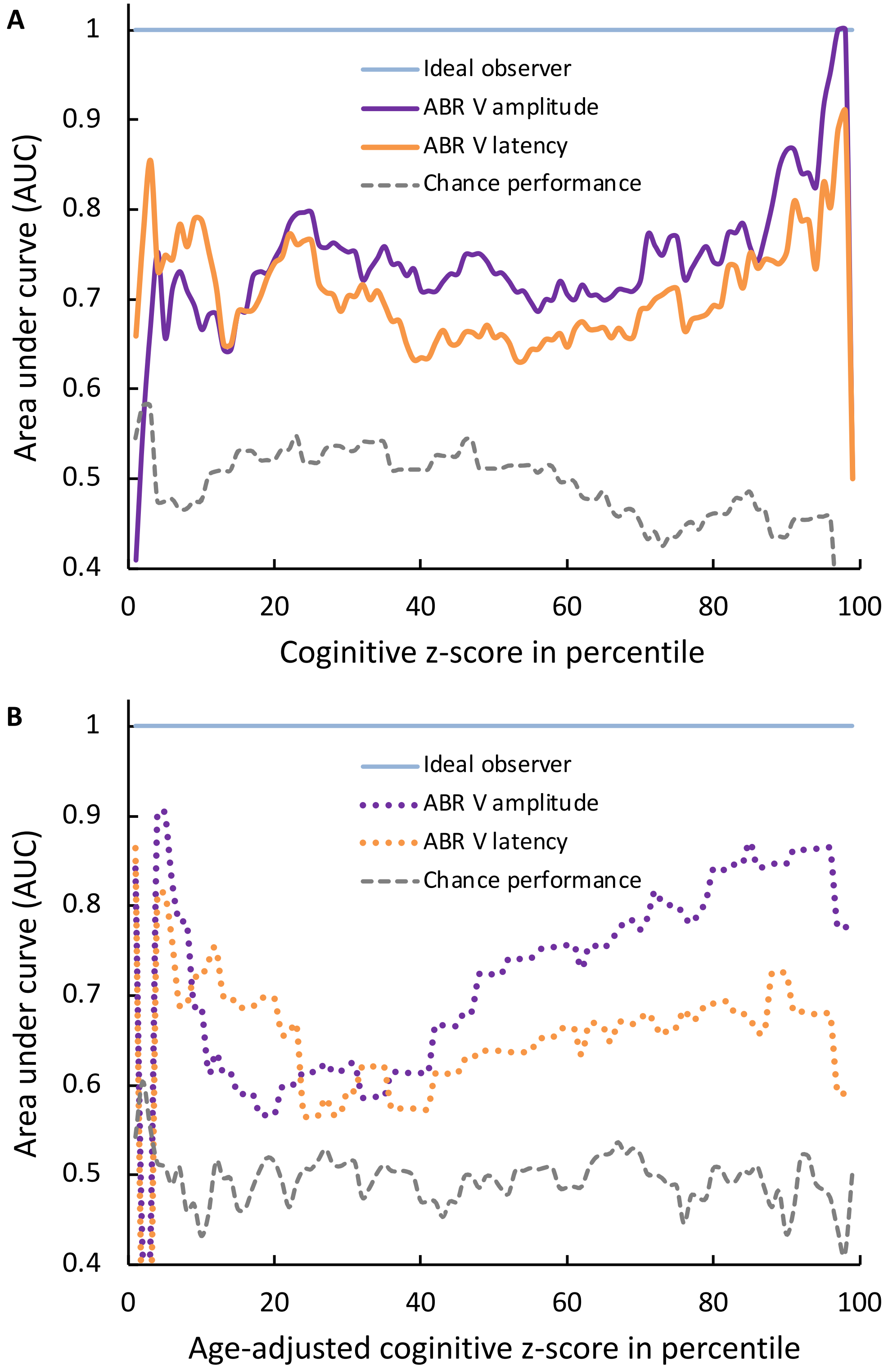

Supplement: Supplementary file 1 — Supplementary Materials [file 42003_2024_7346_MOESM1_ESM.pdf]
