# Supplementary material for: Auditory brainstem responses as a biomarker for cognition
Source: Commun Biol. 2024 Dec 19;7:1653. doi: 10.1038/s42003-024-07346-4 (PMC11659319; doi:10.1038/s42003-024-07346-4)
Supplement: Supplementary file 3 — Description of Additional Supplementary File [file 42003_2024_7346_MOESM3_ESM.pdf]

Description of additional supplementary file

File name: Supplementary data

Description: The source data for Figure 1A, 2, 3, 4 and 5
